# Evaluating reaction norm and fixed effect models for genomic selection of end-use quality in wheat

**DOI:** 10.1007/s00122-026-05394-4

**Published:** 2026-10-05

**Authors:** Jared Lile, Jennifer Lachowiec, Jason D. Fiedler, Jason P. Cook

**Affiliations:** 1 Department of Plant Sciences & Plant Pathology, Montana State University, Bozeman, MT USA; 2 USDA-ARS Cereal Crops Improvement Research Unit, Edward T. Schafer Agricultural Research Center, Fargo, ND USA

## Abstract

***Key message*:**

**Historic breeding program end-use quality data was used to perform genomic selection in two broad frameworks, incorporating fixed effect loci and genotype by environment interactions.**

**Abstract:**

Exceptional end-use quality in hexaploid bread wheat is an important factor for selling wheat in the global marketplace. However, many end-use quality traits are time-consuming and expensive to test, meaning their measurement is often delayed until late-stage trials of a breeding program. Genomic selection (GS) is one tool that has been shown to allow for earlier generation improvement of many traits in wheat breeding. Further, advances in GS methods may improve model predictive abilities, such as incorporating known loci that control traits as fixed effects and utilizing single-stage reaction norm models to control for genotype by environment interactions. Therefore, we evaluated conventional methods as well as these two advances to perform GS for twelve important end-use quality traits collected by the Montana State University (MSU) Spring Wheat Breeding Program from 2010–2022 across 842 elite breeding lines. We found that the conventional GS models performed well in 5-fold cross validation with predictive abilities ranging from 0.4–0.7 and in leave-one-year-out validation with most trait-year combinations exceeding a predictive ability threshold of 0.3. We did not observe any difference in predictive ability for the inclusion of fixed effect loci, but we did observe strong within-year correlations for the single-step reaction norm models in both validation methods. We concluded that both conventional, two-step GS and single-step reaction norm GS offer viable methods for earlier generation improvement of major end-use quality traits in the MSU Spring Wheat Breeding Program.

**Supplementary Information:**

The online version contains supplementary material available at https://doi.org/10.1007/s00122-026-05394-4.

## Introduction

Genomic selection (GS) is a modern breeding technology that has seen wide and diverse adoption over the last decade in both plant and animal breeding (Crossa et al. 2017; Alemu et al. 2024). One primary driver for this adoption is the ability of GS to increase genetic gain over time for many different traits. This is achieved by the incorporation of genome-wide information that can simultaneously model both large and small effect loci, leading to a more comprehensive prediction of trait performance compared to single locus methods like marker assisted selection (MAS), especially for low heritability traits (Bernardo and Yu 2007). Additionally, there have been many recent developments to improve GS models that enable prediction with larger, more complicated multi-environmental datasets.

In hexaploid bread wheat (*Triticum aestivum*), GS has been utilized for numerous traits including yield, grain protein content, and disease resistance (Bonnett et al. 2022; Michel et al. 2019; Steiner et al. 2019; Merrick et al. 2022). One of the major classes of wheat grown in the United States is hard red spring wheat (HRSW) which accounted for 25.5% of the total US wheat production in 2024 (USDA NASS 2024). Hard red wheat, in general, is known for its high grain protein content, gluten strength, and desirable end-use quality which influences its market price (Roberts et al. 2022; Subedi et al. 2023). End-use quality can be broadly split into three categories: milling traits, dough traits, and baking traits. Milling traits refer to the properties and composition of a grain sample and its derived flour, while dough traits and baking traits refer to the properties of the dough and final baked products derived from a flour sample.

End-use quality traits are ideal targets for genomic selection because they are often highly heritable (Souza et al. 2012; Laidig et al. 2017), though also polygenic (Battenfield et al. 2018; Gill et al. 2025). This means end-use quality traits are consistently controlled by genetics, but there are likely too many important loci for a single locus method like MAS to encompass, indicating that a genome-wide method like GS is needed instead. In addition, end-use quality traits are typically measured late in a breeding cycle due to the labor, complexity of tests, and large grain samples required for the measurements (Battenfield et al. 2016). Genomic selection enables end-use quality improvement at earlier stages of the breeding pipeline, increasing the rate of genetic gain over time.

Multiple studies have investigated the use of GS for trait improvement in different wheat classes, leading to several key advances to make better predictions. Two of these advances include incorporating known QTL markers as fixed effects, (Zhao et al. 2014; Michel et al. 2018; Gao et al. 2025) and modeling of genotype by environment (GE) interactions (Burgueño et al. 2012; Sandhu et al. 2022; Brault et al. 2025), though few studies (Albrecht et al. 2024) have incorporated these advances into end-use quality prediction.

End-use quality traits are known to exhibit varying levels of GE (Prasad et al. 2003; Aoun et al. 2021; Shahi et al. 2024) which can lower predictive ability (Burgueño et al. 2012). One method to overcome this is the single-step reaction norm model described in Jarquín et al. (2014) which is an extension of the GBLUP model and performs genomic prediction in one step using the raw phenotypic data as a response. It also models GE interactions between additive genetic values and environmental covariates using prebuilt covariance matrices (Jarquín et al. 2014; Crossa et al. 2017; Persa et al. 2020). This single-step method directly predicts phenotypic values within a specific environment, and it has been successfully utilized in genomic selection for grain yield in wheat (Jarquín et al. 2017; Pérez-Rodríguez et al. 2017).

In this study, we conduct genomic selection for twelve end-use quality traits in the HRSW market class using twelve years of historic quality data from the Montana State University Spring Wheat Breeding Program. We examine two advances in GS: the incorporation of known fixed effect QTL and the modeling of GE interactions with single-step reaction norm models. We utilize two validation schemes (k-fold and leave-one-year-out) to investigate the effectiveness of these methods on their ability to perform genomic selection for end-use quality traits in hard red spring wheat.

## Methods

### Phenotypic data collection

End-use quality data was obtained from the Montana State University Spring Wheat Breeding Program yield trials conducted from the years 2010–2022. Trials were conducted at multiple locations each year, and within each location, two trials consisting of the preliminary yield trial (PYT) and advanced yield trial (AYT) were conducted with different sets of experimental lines and a set of common check varieties (Supplementary Fig. S1). The check varieties also varied between years with some checks consistently included and others replaced over time. Each year, a subset of lines from the PYT were selected to advance to the AYT, resulting in an unbalanced dataset with better performing lines being evaluated in more location-years. Different experimental designs, primarily lattice and randomized complete block, were used over the tested years. One replicate for each line from a trial was submitted for quality testing with one check variety, Duclair, having two replicates for consistency. In total, 842 lines were phenotyped, including 24 check varieties and 818 elite experimental lines from the program. Notably, experimental lines from the 2021 PYT were phenotyped but not genotyped, so that trial only had training data for the check varieties (Supplementary Fig. S1).

End-use quality data was collected by the Montana State University Cereal Quality Laboratory with protocols following the American Association of Cereal Chemists methods (AACC 2000). In brief, moisture and grain protein content were first collected on a cleaned grain sample using a Foss Infratec 1241 Near Infrared Reflectance (NIR) instrument (Infratech 1241 Grain Analyzer; Foss North America) calibrated via AACC Method 46–30 (AACC 2000). The sample was then tempered to 15.5% moisture and milled using a Brabender Quadrumat Sr. Mill (C.W. Brabender Instruments Inc.) with flour yield describing the percentage of pure flour compared to total milled product. After two weeks of stabilization, flour protein was measured using the same Infratec 1241 instrument. Total ash content (wheat ash) and flour ash content were also collected following AACC Method 08–01 adjusted to 14% moisture content (AACC 2000). Dough properties including mix time, mix water absorption, and mix tolerance were collected using a Mixograph following AACC Method 54-40A with a 10g sample corrected to a 14% moisture basis (AACC 2000). Finally, bake quality traits including bake mixing time, bake water absorption, and loaf volume were collected following AACC Method 10-10B (AACC 2000). Single kernel hardness was measured separately via a single-kernel characterization system (SKCS) (Perten Instruments, Springfield, IL, U.S.A.).

Broad-sense heritability was calculated for each end-use quality trait using the Cullis method with the following formula:

1 $${H}_{Cullis}^{2} = 1 - \frac{{\overline{v} }_{\Delta }^{BLUP}}{2 * {\sigma}_{g}^{2}}$$

where ${\sigma}_{g}^{2}$ is the genotypic variance and ${\overline{v} }_{\Delta }^{BLUP}$ is the mean variance of a difference between two genotypic Best Linear Unbiased Predictors (BLUPs) (Cullis et al. 2006). Both ${\sigma}_{g}^{2}$ and BLUP values were estimated from the model described in Eq. 2 except that ${Line}_{i}$ was treated as a random effect. The Cullis method was selected due its performance in unbalanced datasets (Schmidt et al. 2019) which was the case in this study as the check varieties had many more observations than the elite lines.

### Genetic data collection

Genotyping-by-sequencing (GBS) was performed on the 842 phenotyped lines at the USDA-ARS Genotyping Lab (Fargo, North Dakota) following a protocol adapted from Poland et al. (2012), which uses the double restrictions enzymes, *Pst*I and *Mse*I. GBS libraries were sequenced with Illumina NextSeq 2000 system with 150 bp paired ends (Illumina, San Diego, USA). Sequence data were quality filtered and trimmed for adapters with *bbduk* (BBTools, https://jgi.doe.gov/data-and-tools/bbtools). The trimmed paired-end sequences were aligned to the Chinese Spring wheat IWGSC RefSeq v1.0 reference genome (IWGSC et al. 2018) using Bowtie2 (Langmead and Salzberg 2012). The aligned reads were then sorted and indexed using SAMtools (Danecek et al. 2011, 2021), and vcf files were generated with mapping quality score of at least 30, using BCFtools (Danecek et al. 2021). The variant file was further filtered using vcftools, with a max-missing of 50%, a minor allele frequency (MAF) of 0.01, and a minimum depth coverage of 4. Chromosome UN was removed, and missing data was imputed using Beagle v5.4 (Browning et al. 2018) using default settings, no reference panel, and the Synthetic W7984 x Opata M85 RIL 906 genetic map (Gutierrez-Gonzalez et al. 2019) to inform recombination frequencies.

In total, there were 21,958 SNPs after filtering and imputation. Principal component analysis (PCA) was performed on the imputed numeric SNP matrix using the prcomp R function with scaling and centering to investigate the relatedness between genotyped lines (Supplementary Fig. S2). All SNPs were used for all genomic selection models. For genome wide association studies (GWAS), SNPs with a MAF less than 0.01, which may have been introduced by imputation, were removed.

### Two-step analysis method: first step

Two methods of genomic prediction were utilized in this study. The first method, hereafter the “two-step method” (Garrick et al. 2009), is an approach where the analysis is split into two separate steps. In the first step, summary values for each phenotyped line are calculated across all environments. Then, in the second step, the summary values are used as the response variable in a new model with the genetic markers to calculate genomic estimated breeding values (GEBVs). In this study, best linear unbiased estimators (BLUEs) (Holland and Piepho 2024) were calculated for each line from the following model:

2 $$\begin{aligned}{Trait}_{ijk}&= \mu + {Line}_{i}+{Environment}_{j}\\&\quad +{Line*Environment}_{ij}+ {\varepsilon}_{ijk} \end{aligned}$$

where *Trait*_*ijk*_ is the *k* ^th^ observation of an end-use quality trait measured on the *i* ^th^ line in the *j* ^th^ environment where an environment denotes a single combination of a year, location, and trial (AYT or PYT). ${Line}_{i}$ is fixed effect of the *i* ^th^ line, ${Environment}_{j}$ is the random effect of *j* ^th^ environment following a $\mathcal{N}\left(0,{\sigma}_{Env}^{2}\right)$ distribution, ${Line*Environment}_{ij}$ is the random interaction of the *i* ^th^ line with the *j* ^th^ environment following a $\mathcal{N}\left(0,{\sigma}_{Line * Env}^{2}\right)$ distribution, and ${\varepsilon}_{ijk}$ is the random error term following $\mathcal{N}\left(0,{\upsigma }^{2}\right)$. These models were fit using the lme4 package (Bates et al. 2015), and BLUEs were extracted using the emmeans package (Lenth 2024) in the data analysis software R (R Core Team 2024).

### Two-step analysis method: second step baseline

The second step of the analysis was to fit standard genomic selection models including RR-BLUP with the rrBLUP R package (Endelman 2011) and Bayesian Ridge Regression (BRR), Bayesian LASSO (B.Lasso), BayesA, BayesB, and BayesC models using the BGLR R package (Pérez and de los Campos 2014). Reproducing kernel Hilbert space (RKHS) models were also fit with the BGLR package using a Gaussian kernel computed as follows:

3 $$\mathbf{K}\left({x}_{i},{x}_{j}\right)=\mathrm{exp}\left(-h\left(\frac{{d}_{ij}}{k}\right)\right)$$

where ${d}_{ij}$ is the squared Euclidean distance based on genetic markers between individuals *i* and *j*, *k* is the mean squared Euclidean distance across all individuals, and *h* is a bandwidth parameter set equal to one. All Bayesian models were fit using 10,000 iterations, with the first 1,000 used as burn-in and the default thinning value of 5.

### Two-step analysis method: second step GWAS

Genome wide association studies (GWAS) were conducted for each end-use quality trait using GAPIT version 3 (Wang and Zhang 2021) with the BLINK model (Huang et al. 2019). A MAF threshold of 0.01 for SNP inclusion was used for all GWAS, as well as two principal components to control for population structure. Significant markers for each trait were determined using the default Bonferroni correction thresholds (Wang and Zhang 2021) (Supplementary Table S1). GWAS-informed genomic selection models (Bernardo 2014) were identical to the models described above except that the significant GWAS markers for a given trait were removed from the random effect marker matrix and treated instead as unique fixed effect variables. During cross-validation, GWAS was performed using only the training data in each fold to avoid so called “inside trading” effect of using fixed GWAS markers identified with the full phenotypic dataset (Arruda et al. 2016). To illustrate this effect, separate models were tested with the inclusion of GWAS markers identified on the full dataset.

### Two-step analysis method: second step known QTL

Genes corresponding to major wheat seed proteins including the glutenin, gliadin, puroindoline, purothionin, avenin-like, purinin, and triticin families, as well as amylase/trypsin inhibitors, were considered known loci that could potentially influence end-use quality (Juhász et al. 2018; Zhang et al. 2018; Li et al. 2020). Markers were first filtered to those that fell within 1 Mb of any known seed protein genes, and in cases where multiple markers were close to a gene, the marker with the highest minor allele frequency was selected. Selected markers were treated as fixed effects in two-step GS models in the same manner as the GWAS-informed models above.

In addition to selecting markers based on minor allele frequency, we also selected all genetic markers within 1 Mb of any known seed protein gene and performed principal component analysis on the resulting marker matrix. Two-step GS models were then cross-validated with the inclusion of the first 1, 3, 20, or 50 principal components as fixed effects, with the selected markers removed from the random effect marker matrix as before.

### Single-step analysis method

The second method of genomic prediction used in this study is hereafter referred to as the “single-step” method, which combines the raw phenotypic data and genetic marker data together into one prediction model. These models were based on the reaction norm models described in Jarquín et al. (2014) and can be generally written as follows:

4 $${y}_{ijk}= \mu +{E}_{i}+ {L}_{j}+{EL}_{ij}+{\epsilon}_{ijk}$$

where ${y}_{ijk}$ is the *k* ^th^ observation of an end-use quality trait measured on the *j* ^th^ line in the *i* ^th^ environment where, as before, an environment denotes a combination of a year, location, and trial within a location (AYT or PYT). *μ* is the overall trait mean, ${E}_{i}$ is the fixed effect of the *i* ^th^ environment, and ${L}_{j}$ is the random effect of the *j* ^th^ line with $\mathbf{L} \sim \mathcal{N}\left(0,{\mathbf{Z}}_{\mathrm{L}}\mathbf{K}{\mathbf{Z}}_{\mathrm{L}}^{^{\prime}}{\sigma}_{Line}^{2}\right)$. ${EL}_{ij}$ is the interaction between the *i* ^th^ environment and the *j* ^th^ line with $\mathbf{E}\mathbf{L} \sim \mathcal{N}\left(0,\left[{\mathbf{Z}}_{\mathrm{L}}\mathbf{K}{\mathbf{Z}}_{\mathrm{L}}^{^{\prime}}\right]{\circ \left[{\mathbf{Z}}_{\mathrm{E}}{\mathbf{Z}}_{\mathrm{E}}^{^{\prime}}\right] \sigma }_{EL}^{2}\right)$ where *∘* is the Hadamard product, ${\mathbf{Z}}_{\mathrm{L}}$ is an $\left(n\times j\right)$ incidence matrix for phenotypic observations of each line, ${\mathbf{Z}}_{\mathrm{E}}$ is an $\left(n\times i\right)$ incidence matrix relating observations to an environment, $\mathbf{K}$ is either the $\left(j\times j\right)$ Gaussian kernel matrix described in Eq. 3 for the RKHS models, or for all other genomic selection models, $\mathbf{K}$ is the $\left(j\times j\right)$ genomic relationship matrix calculated using the *a.mat* function from the rrBLUP R package (Endelman 2011) using the GBS markers. Finally, ${\epsilon}_{ijk}$ is the random error term following ${\epsilon}_{ijk} \sim i.i.d. \mathcal{N}\left(0,{\upsigma }^{2}\right)$. Code to implement these models was based on the framework provided in Montesinos-López et al. 2021.

### Cross-validation

Two methods of cross-validation were used to assess the predictive ability of tested models. The first method was a five-fold cross-validation which differed slightly in implementation between the two-step and single-step methods. In the two-step method, the line BLUEs were randomly divided into five equal folds and each fold was sequentially removed and then predicted using the remaining folds of data. This procedure was repeated using the same folds each time to evaluate all of the candidate GS models. Pearson’s correlation between the predicted and observed values within a fold was measured and reported as the model’s predictive ability. The root mean squared error (RMSE) and mean average error (MAE) were also recorded (Supplementary Table S2). Twenty allocations of five folds were created for a total of 100 cycles of cross-validation for each model. The mean correlation, RMSE, and MAE were taken across all cycles as well as the standard deviation of the correlations to give a sense of the range of values between cycles.

For five-fold cross-validation of the single-step method, the raw phenotypic data points at the observation level were randomly assigned into five equal folds. One fold was then removed and the candidate model was used to directly predict the removed phenotypic values. The correlation, RMSE, and MAE were taken both at the location-year level and across all location-years (Supplementary Table S3). Both methods were then averaged across 100 cycles of cross-validation for all metrics, and the standard deviation of the correlation values was also taken.

The second method of cross-validation was a leave-one-year-out (LOYO) validation. For the two-step approach, all non-check lines phenotyped in a given year were identified and their BLUEs were removed. The remaining lines were then used to predict GEBVs for the removed lines, and the correlation, RMSE, and MAE were calculated (Supplementary Table S4). The removal of the most recent year in the dataset (2022) was technically a forward prediction as all previous years of data were used to predict the newest year. Similar methods were used for the single-step approach, but specifically the phenotypic values of non-check lines within a year were removed, while the check lines were retained. This represented a “CV2” style of prediction (Jarquín et al. 2017) in which a breeder could phenotype only a small number of core “check” lines from a certain environment and then predict the phenotypes for all of the experimental lines in that same environment. The metrics correlation, RMSE, and MAE were taken both at the location-trial level within a year and across all locations (Supplementary Table S5).

## Results

**Fig. 1:**
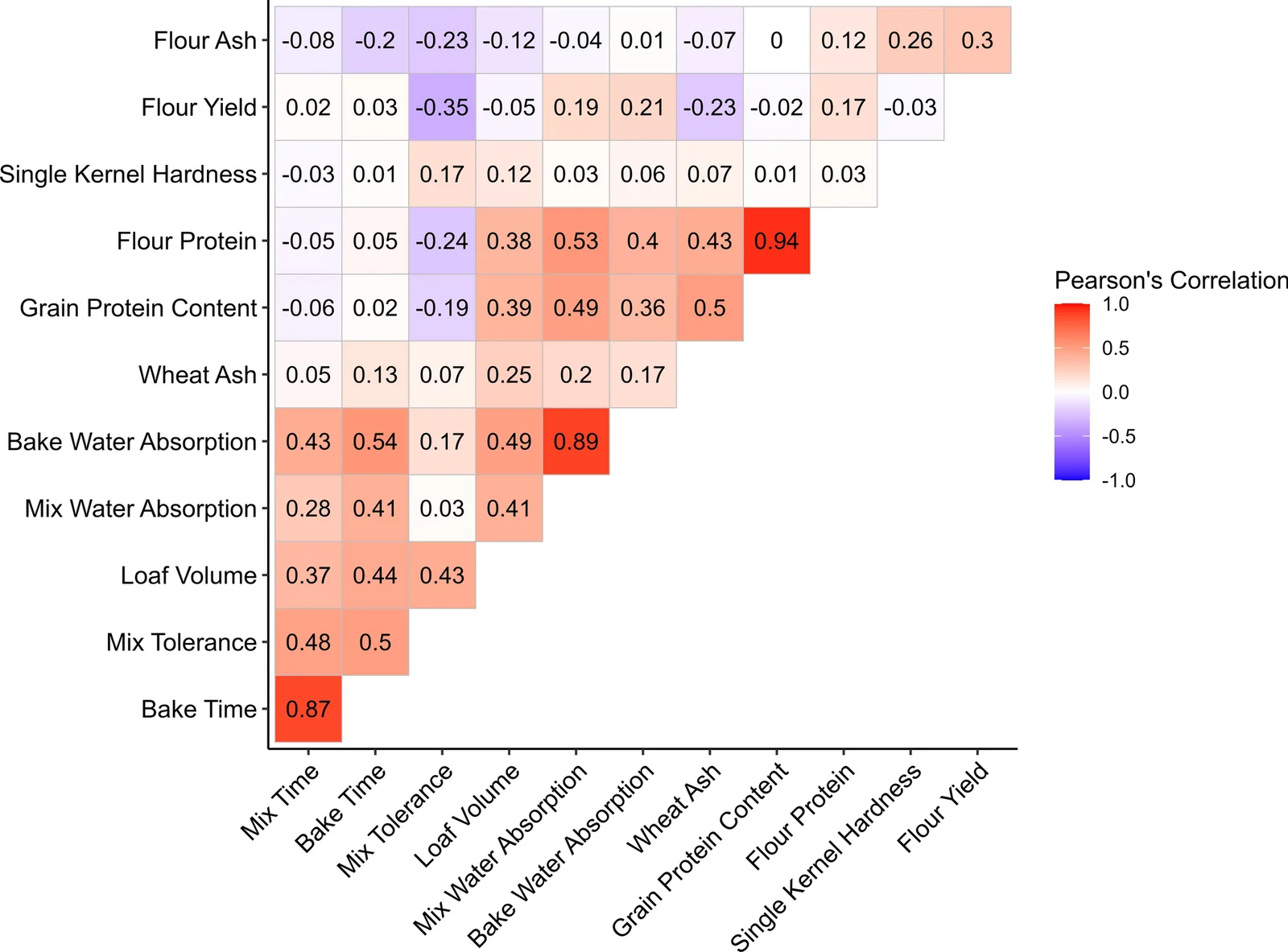
Pearson correlation matrix between the BLUEs of the twelve end-use quality traits with darker red indicating a stronger, positive correlation and darker blue indicating a stronger, negative correlation

Predictive ability in genomic selection (GS) depends on, among other factors, the heritability of the predicted trait (Bernardo 2020). Broad-sense heritability for the twelve end-use quality traits tested in this study was determined (Table 1) using the Cullis method (Cullis et al. 2006). Wheat ash content had the lowest heritability with a value of 0.39, with all other traits exceeding a broad-sense heritability of 0.60. Pairwise Pearson correlation coefficients between the BLUEs of all 12 end-use quality traits were also calculated (Fig. 1). The pairs of traits with high correlation (above 0.85) were flour protein content and grain protein content, bake water absorption and mix water absorption, and bake time and mix time. Grain protein content is commonly used as an indicator proxy trait for overall end-use quality but was only moderately, positively correlated with four out of the eleven traits and strongly correlated only with flour protein content.

**Table 1:** Broad-sense heritabilities for twelve end-use quality traits calculated using the Cullis method

Trait	H^2^ Cullis
Single kernel hardness	0.89
Bake time	0.87
Mix time	0.84
Loaf volume	0.81
Flour yield	0.79
Mix tolerance	0.76
Flour ash content	0.75
Bake absorption	0.73
Grain protein content	0.70
Flour protein	0.69
Mix absorption	0.63
Wheat ash content	0.39

### Two-step analysis results

**Fig. 2:**
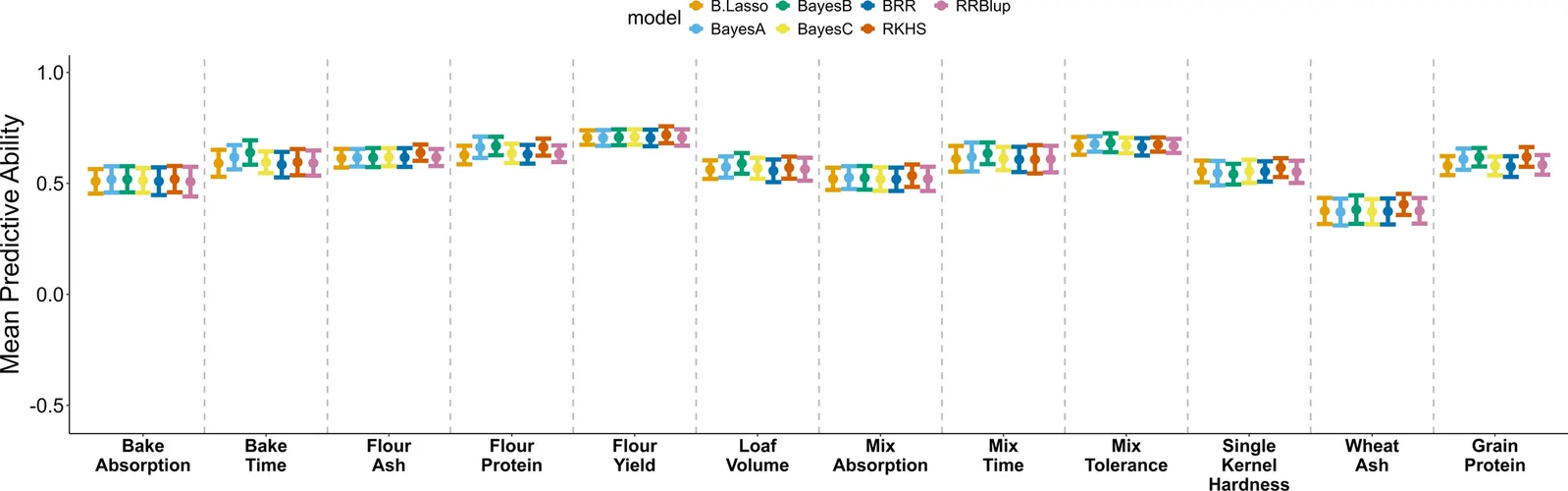
Five-fold cross-validation results (n=100) for twelve end-use quality traits. The mean predictive ability is given on the y-axis and error bars denote one standard deviation. Colors represent results from one of seven different genomic selections that were tested

Seven genomic selection models were tested for each end-use quality trait as the second stage of the two-step analysis. One hundred cycles of five-fold cross-validation for each model within each quality trait were performed (Fig. 2). The mean predictive ability across all cycles ranged from 0.4 to 0.7 with standard deviations between 0.04 to 0.06. There was no appreciable difference between the mean predictive abilities of the different genomic selection models within each trait. However, predictive ability did vary across traits with mix tolerance and flour yield having the highest predictive ability on average, and the trait with the lowest heritability, wheat ash content, having the lowest predictive ability.

**Fig. 3:**
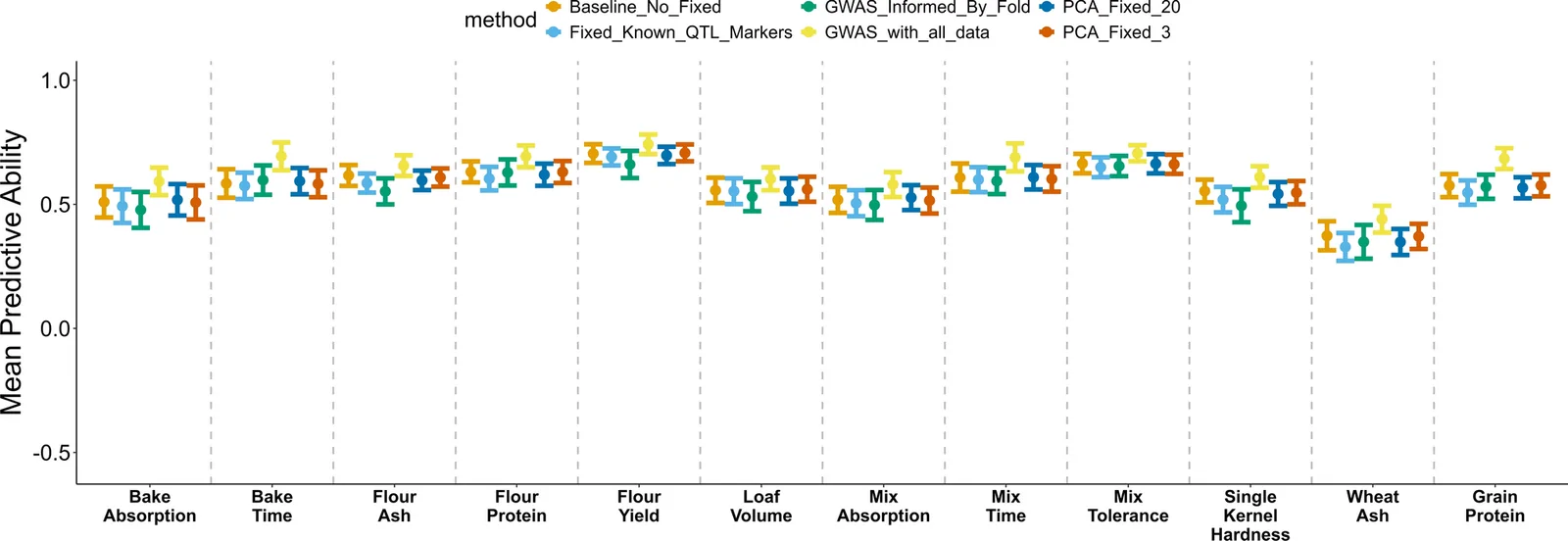
Five-fold cross-validation results (n=100) for the two-step method showing the mean predictive ability for twelve different end-use quality traits. The BRR genomic selection model was used in all cases with different colors representing different fixed effect specifications. The first, orange bars are the baseline model with no added fixed effect terms which are compared against the inclusion of markers associated to potential known QTL, markers identified by GWAS on each training fold, markers identified by GWAS on the full phenotypic BLUEs dataset, and 20 or 3 principal components of a PCA across all markers associated with a potential known QTL

Given that most of the end-use quality traits had a high broad-sense heritability (Table 1) and there are known major genes controlling them (Eagles et al. 2002), we next investigated the effect of removing certain markers from the random effect marker matrix and treating them as fixed effects to exert a stronger influence on trait predictions. Using the BRR genomic selection model within the two-step method framework for consistency, we found no difference in the average predictive ability, across 100 cycles of five-fold cross-validation, between the baseline model with no added fixed effects compared to models with included fixed effect markers (Fig. 3). The only case where an increase in mean predictive ability was observed, around one standard deviation higher for all traits, was when we included fixed effect markers identified by a GWAS analysis of the full phenotypic BLUEs dataset (Fig. 3). This method problematically included both training and validation sets of data, which led to data leakage and inflated predictive abilities (Supplementary Table S2).

**Fig. 4:**
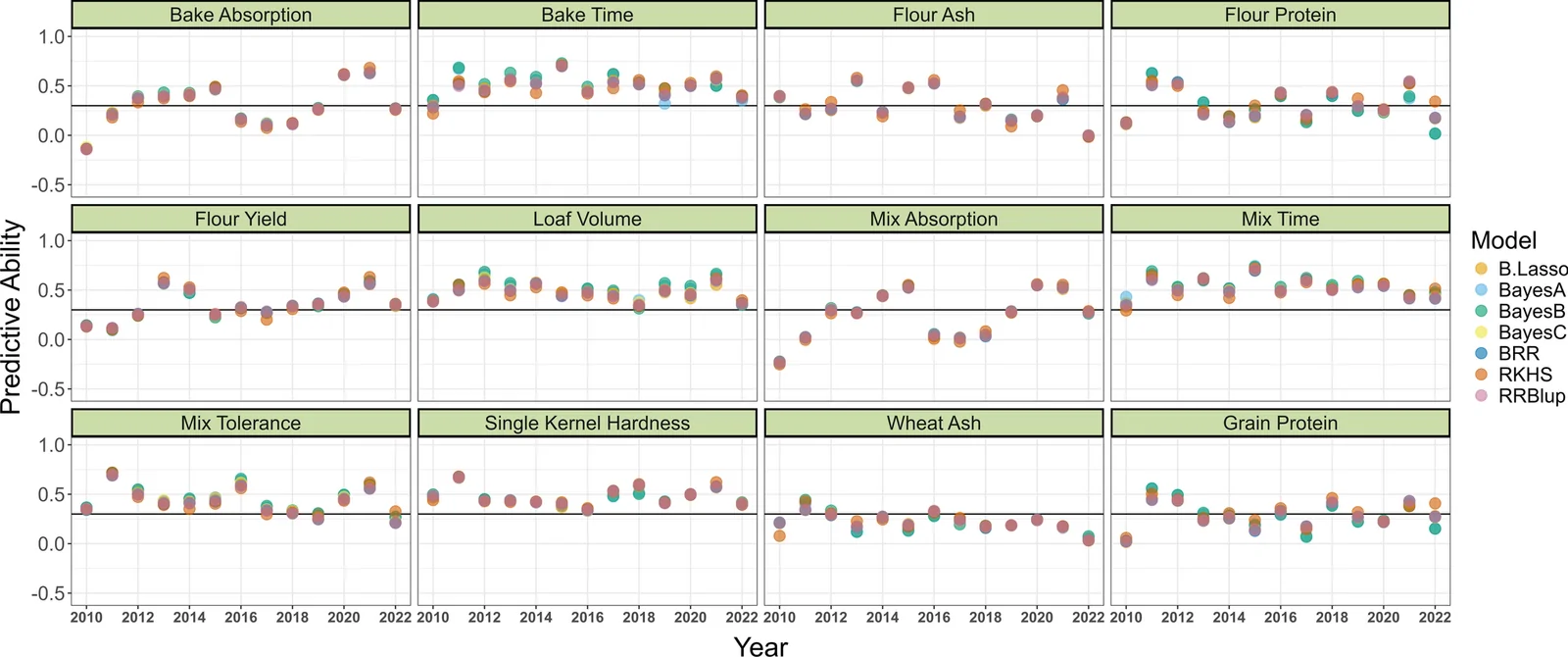
Leave-one-year-out (LOYO) validation results for the two-step analysis approach. The x-axis denotes the year in which the experimental lines were left out of the training data and predicted. The y-axis shows the predictive ability as the correlation between the predicted breeding values and known BLUEs for the experimental lines in the left-out year. Each box represents one end-use quality trait, and for each trait, results are given for seven different GS models denoted with different colors

Leave-One-Year-Out (LOYO) validation was performed for the same seven GS models as the five-fold cross-validation (Fig. 4). A cutoff of 0.3 was used to delineate a useful prediction versus an uninformative or even detrimental one (Heffner et al. 2010; Marulanda et al. 2021). The traits bake time, loaf volume, mix time, mix tolerance, and single kernel hardness had predictive abilities above the 0.3 cutoff for all 13 years. Other traits varied in predictive ability depending on the year left out, and wheat ash content was the only trait that consistently failed to meet the cutoff for most years. For all traits, performance of different models was nearly identical, though notably, in the forward prediction year 2022, the traits grain protein content and flour protein content had discernably higher predictive abilities for the RKHS model compared to all other models.

### Single-step analysis results

**Fig. 5:**
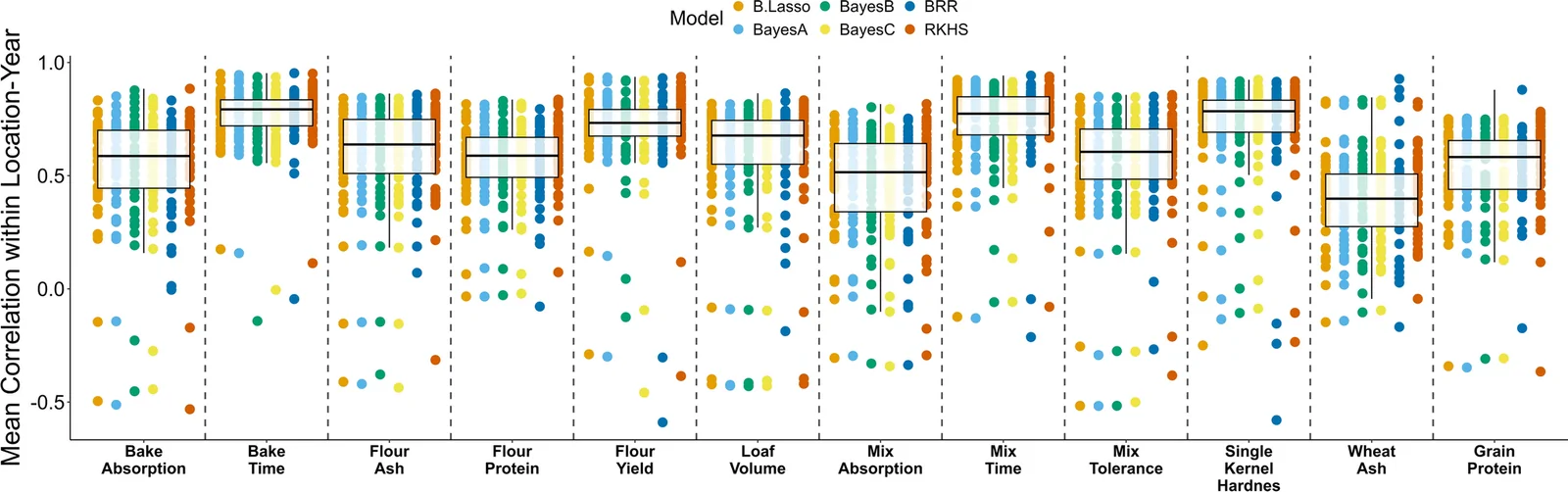
Five-fold cross-validation results (n=100) for the single-step analysis method for twelve end-use quality traits. The mean predictive ability across all cycles of cross-validation, within a location-year, represented by each point. The x-axis denotes the quality trait tested and each color represents a different genomic selection model. Box plots show the overall median of the means predictive ability for each trait across all location-years and models

Five-fold cross-validation for the same six GS models (without RR-BLUP, see Methods) was also performed with the single-step analysis method (Fig. 5). Predictive ability in this context refers to the correlation between observed phenotypic values and predicted phenotypic values within a location-year combination across the preliminary and advanced yield trials conducted at that site. The mean predictive ability refers to the average correlation within a location-year across 100 cycles of cross-validation. There was little discernible difference between the different models tested. The traits with the highest median of the means predictive ability across all models and location-years were bake time, flour yield, mix time, and single kernel hardness with values at nearly 0.8, while the trait with the lowest median predictive ability was wheat ash content at a little under 0.5. Interestingly, these results corresponded almost exactly to the broad-sense heritabilities (Table 1) in terms of the relative trait rankings.

**Fig. 6:**
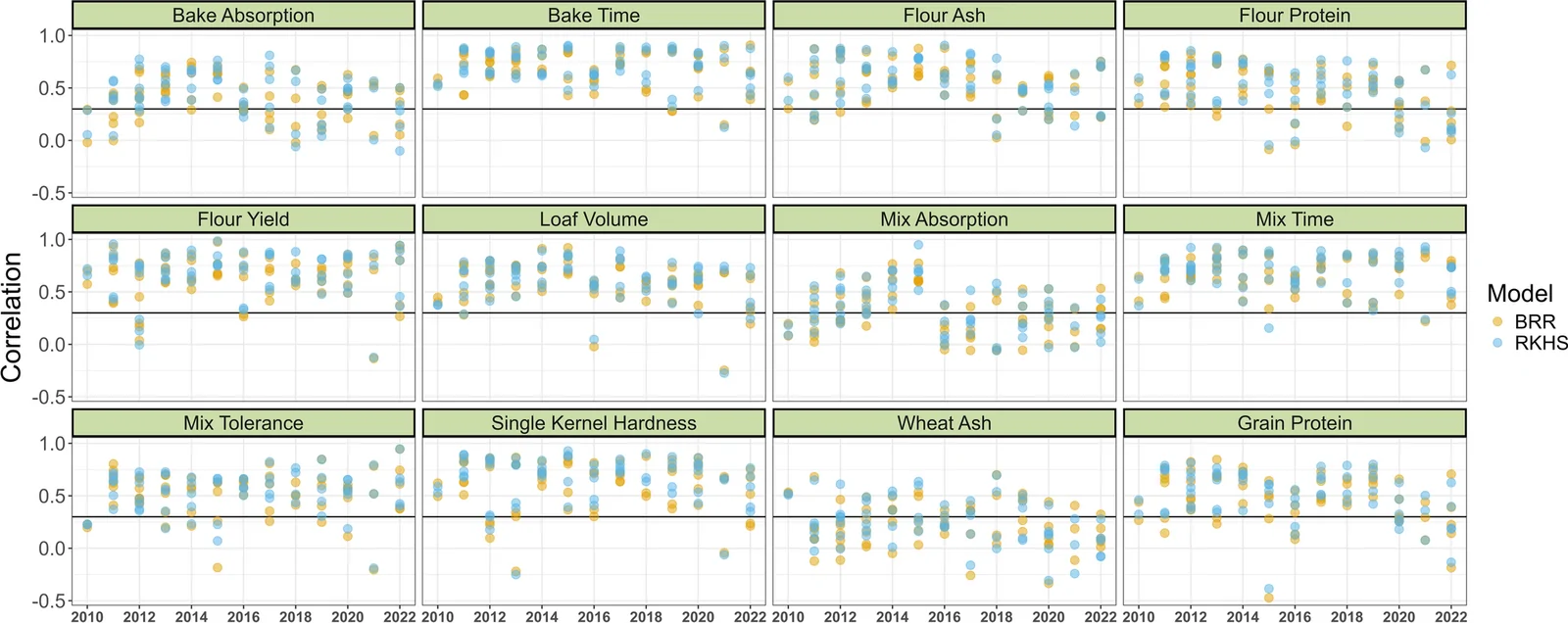
Leave-one-year-out (LOYO) validation results for the single-step method of analysis. The correlation between predicted and observed phenotypic values within a location-trial are shown on the y-axis with points representing one location-trial combination within the left-out year, denoted by the x-axis. Two representative genomic selection models (BRR and RKHS) are shown in different colors

Leave-One-Year-Out (LOYO) validation was conducted on the single-step analysis method in the same manner as the two-step except that predictive ability was taken as the correlation between predicted and observed phenotypic values within a location- trial combination for each within the left-out year (Fig. 6). As before, a minimum predictive ability of 0.3 was considered the threshold for a useful model. Traits such as mix time and bake time had very consistent predictive ability between different location-trials within the same year. For all traits however, this consistency varied somewhat between years (Fig. 6).

## Discussion

The goal of this project was to perform genomic selection (GS) for end-use quality traits in hard red spring wheat. Two broad approaches for GS were tested: the two-step method in which summary BLUEs were precalculated before performing GS and the single-step method in which genetic markers were used in tandem with raw phenotypic data. Both methods were tested under two types of cross-validation, five-fold and Leave-One-Year-Out (LOYO). Further, within the five-fold cross-validation of the two-step method, the inclusion of different types of fixed effects was assessed.

Broad-sense heritabilities for the twelve end-use quality traits were generally high, ranging from 0.4 to 0.9 (Table 1) which was consistent with expectations and matched well with other studies in bread wheat that tested overlapping traits with the same method of heritability calculation (Sandhu et al. 2022; Gill et al. 2023). Correlations between trait BLUEs revealed that they could be sorted into roughly three (overlapping) groups with group one consisting of mix time, bake time, mix tolerance, loaf volume, mix water absorption, and bake water absorption; group two consisting of loaf volume, mix water absorption, bake water absorption, total wheat ash content, grain protein, and flour protein; and group 3 consisting of flour ash content, flour yield, and single kernel hardness (Fig. 1). Most notably, grain protein content, which is widely used as an indicator of overall quality, was only positively correlated with five out of eleven traits and was weakly, negatively correlated with the important gluten strength trait, mix tolerance (Fig. 1). This was also consistent with the literature as many studies have shown that grain protein content is uncorrelated with other important quality traits such mix time, bake time, flour yield, and gluten strength (Battenfield et al. 2016; Ibba et al. 2020; Zhang-Biehn et al. 2021; Sandhu et al. 2021; Gill et al. 2025) showing that predicting these traits directly with GS could be a superior method of improving them compared to selecting based on protein content.

The two-step method showed promising results for all quality traits except for total wheat ash content which, consistent with its lower broad-sense heritability, under performed in both cross-validation methods (Figs. 2 and 4). Mean predictive abilities in the five-fold cross-validation for all the other traits met or exceeded 0.5 (Fig. 2) which was 0.1–0.2 units higher than the k-fold validation results for the same models and traits reported in some studies (Zhang-Biehn et al. 2021; Fradgley et al. 2023; Gill et al. 2023) though consistent with the results from other studies (Battenfield et al. 2016; Sandhu et al. 2022). This was not surprising as the training dataset used in this study was large relative to the size of the breeding program. Predictive ability is expected to increase as the training population size increases and as the relatedness between lines increases (Bernardo 2020). The individuals in a smaller breeding program, such as the one here, are likely to be more closely related than those from larger, more diverse programs. We demonstrated this expectation via a principal component analysis on the genetic marker data which showed no signs of significant population structure or clustering (Supplementary Fig. S2). Further, twelve years of multi-environmental training data is substantial, and nearly 22,000 genetic markers would be expected to provide excellent, whole-genome coverage. All of these factors in tandem suggest that somewhat higher predictive abilities, compared to some other studies, would be observed.

Additionally, an updated version (v2.1) of the IWGSC Chinese Spring reference genome has been released (Zhu et al. 2021). Using this version for sequence alignment in future genotyping protocols could result in fewer unmapped reads and thus superior marker imputations and a greater number of available genetic markers for model training, which in turn could also increase predictive ability (Solberg et al. 2008).

In the more rigorous LOYO validation, most quality traits had a predictive ability at or above the threshold for successful predictions of 0.3 for all years (Fig. 4). The exception being wheat ash content, and to a lesser extent, mix water absorption. This indicated that the majority of end-use quality traits could be consistently predicted with a useful level of predictive ability in spite of the year-to-year variation. Also of note, in the case of two-step methods only, the model prediction accuracy (correlation between predicted breeding values and true breeding values) would likely be higher than the reported predictive abilities, especially for the traits like wheat ash content that had lower heritability (Table 1; Wang et al. 2025).

Interestingly, the forward predictions, which correspond to the most recent year, 2022, in the LOYO figure, were generally equal to or less than the values for the same trait-model forward predictions reported in other studies (Battenfield et al. 2016; Gill et al. 2025). The results from Gill et al. (2025) are especially of note as, to our knowledge, this is the only other study to investigate genomic selection of end-use quality traits within the hard red spring wheat market class. While our five-fold validation results tended to be equal or higher than their ten-fold validation results, our forward predictions were generally quite similar. A driving reason for this was likely the lack of available genotypic data for the 2021 PYT lines, which meant that the number of training lines in the 2022 AYT was quite small, only consisting of lines from two or more years prior and the usual check varieties. The AYT lines having phenotypic data from previous years surely helped the model predict the AYT and PYT lines in a new year, and the reduction in the AYT sample size would likely reduce the overall predictive ability.

End-use quality in hexaploid bread wheat is known to be controlled by several major QTL (Payne 1987; Eagles et al. 2002; Rai and Han 2023); further, it has been shown in wheat that giving markers associated with major QTL more weight in a genomic selection model, such as by treating them as fixed effects, can improve predictive ability (Rutkoski et al. 2014; Zhao et al. 2014; Bentley et al. 2014; Albrecht et al. 2024). However, the method of identifying which markers to treat as fixed is also important. Notably, including markers that were identified by association mapping on the combined training and testing datasets for GS can lead to artificially inflated predictive abilities in a phenomenon termed the “inside trading effect” (Arruda et al. 2016). We demonstrated this effect in the five-fold cross-validation of the two-step method in which we observed a clear increase in predictive ability when markers detected by GWAS on the full BLUEs dataset were treated as fixed effects (Fig. 3), similar to findings in Michel et al. (2018). Additionally, Michel et al. (2018) found a slight increase in predictive ability when markers associated with known high molecular weight glutenin genes were treated as fixed effects, which avoids the inside trading issue. Building on this, we identified genetic markers associated with the known major seed protein genes and treated those markers as fixed effects. Because many of these genes had multiple markers within 1 Mb, in addition to selecting just one marker per gene, we also performed principal component analysis on all candidate markers and then included different numbers of principal components as fixed effects in GS models. Finally, we incorporated de novo GWAS markers identified by performing GWAS on each training fold of cross-validation rather than the full dataset as fixed effects (Spindel et al. 2016). We did not find a discernable improvement to predictive ability with any of these approaches, which suggested that other methods of improving model predictions should be explored in this population.

Genotype by environment (GE) interactions can reduce predictive ability and are known to be present in many end-use quality traits (Aoun et al. 2021). One method to account for these interactions is the single-step reaction norm model described in Jarquín et al. (2014). We investigated if these GE models could be used to reliably predict end-use quality phenotypes within specific environments. This would mimic the case where only a small number of check varieties could be phenotyped from a trial, and the model would be used to predict the phenotypes of the remaining untested experimental lines. The median of the predictive abilities across all location-years tended to be quite high, corresponding very closely to the broad-sense heritability values for the respective quality trait (Table 1, Fig. 5). The LOYO validation showed consistently strong performance for the traits mix tolerance, mix time, bake time, flour yield, and loaf volume, though other traits including grain protein, flour protein, wheat ash, and mix absorption had lower and less consistent predictive abilities (Fig. 6). Further, both validation approaches had variability in predictive ability between different environments, with a few having notably lower predictive abilities than the others (Figs. 5 and 6). This was probably in part due to the same issue with the 2021 PYT lines discussed in the two-step section. However, it also is possible that there are some outlier environments in the training data, and optimization of the training data by removing any outlier environments could lead to higher and more consistent predictive ability (Heslot et al. 2013; Aoun et al. 2021).

The results from the two-step and single-step methods are not directly comparable in this study as the two-step method predicted genomic estimated breeding values (GEBVs) for each line whereas the single-step method was used to directly predict phenotypic values for a line grown in a specific environment. Both outputs could be of use to a breeder as the GEBV represents the performance of a line as a potential parent across all tested environments whereas the single-step method gives the predicted performance for a line itself within a specific environment of interest (Jarquín et al. 2017; Persa et al. 2020). Both methods performed well; specifically, in the LOYO validation, both methods consistently gave predictive abilities above the threshold of 0.3 (Figs. 4 and 6) for the majority of traits.

We showed that two methods of genomic selection, two-step BLUEs and single-step reaction norm models with a marker by environment interaction term, could be successfully used to predict important end-use quality traits in the hard red spring wheat breeding program at Montana State University. We expect that implementing these methods will allow for improvement of many end-use quality traits at earlier stages in the breeding program before phenotyping is possible. This would increase the selection intensity for these traits and lead to increased genetic gain over time for end-use quality; ensuring that breeding targets for these traits are met and that important markets, which depend on quality standards, will be maintained for producers in years to come.

## Supplementary Information

Below is the link to the electronic supplementary material.

Supplementary file1

Supplementary file2

## Data Availability

The genotypic data, phenotypic datasets, and R code scripts used to generate the results in this study are publicly available on GitHub at the following link: https://github.com/Jslile/Evaluating-Reaction-Norm-and-Fixed-Effect-Models-for-Genomic-Selection-of-End-Use-Quality-in-Wheat.
