# Supplementary material for: Evaluating reaction norm and fixed effect models for genomic selection of end-use quality in wheat
Source: Theor Appl Genet. 2026 Oct 5;139(10):293. doi: 10.1007/s00122-026-05394-4 (PMC13638722; doi:10.1007/s00122-026-05394-4)
Supplement: Supplementary file 1 [file 122_2026_5394_MOESM1_ESM.pdf]

Evaluating Reaction Norm and Fixed Effect Models for Genomic Selection of End-Use Quality in Wheat

Theoretical and Applied Genetics

Supplementary Materials

Jared Lile[1*], Jennifer Lachowiec[1], Jason D. Fiedler[2], Jason P. Cook[1*]

1. Department of Plant Sciences & Plant Pathology, Montana State University, Bozeman, MT, USA

2. USDA-ARS Cereal Crops Improvement Research Unit, Edward T. Schafer Agricultural Research Center, Fargo, ND, USA

*Corresponding authors: jared.lile@student.montana.edu; jason.cook3@montana.edu

Table of Contents

Supplementary Figure S1 3

Supplementary Figure S2 4

Supplementary Tables S1-S5 .... Excel Files

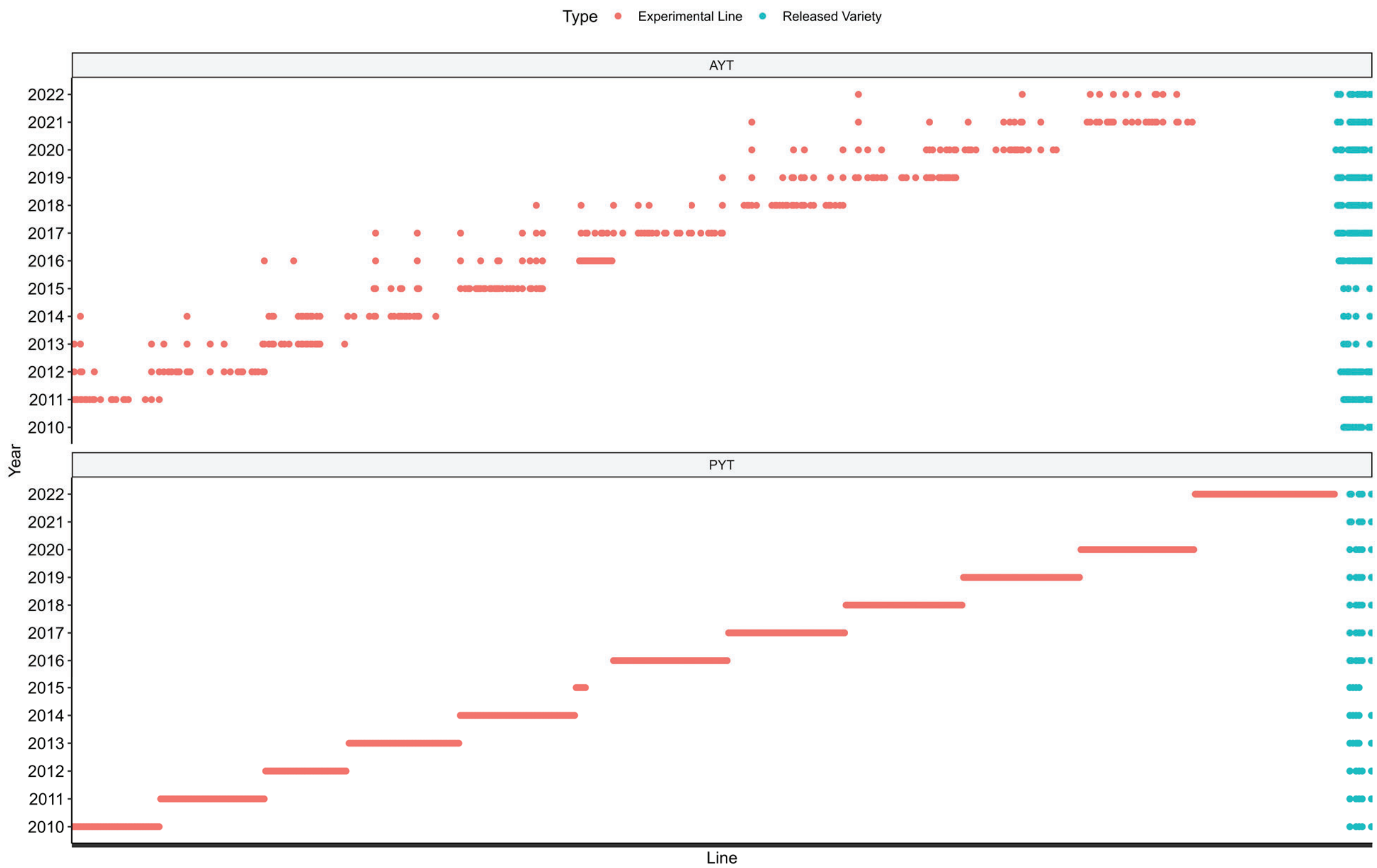

Supplementary Figure 1 Index plot showing the number of experimental lines (red) and released varieties (blue) present in each advanced yield trial (AYT) and preliminary yield trial (PYT) that were both genotyped and phenotyped for end-use quality traits. The x-axis represents each line used in this study, and the y-axis indicates which years a given line was observed. Notably, there were no experimental lines from the 2021 PYT due to a lack of available genotypic information.

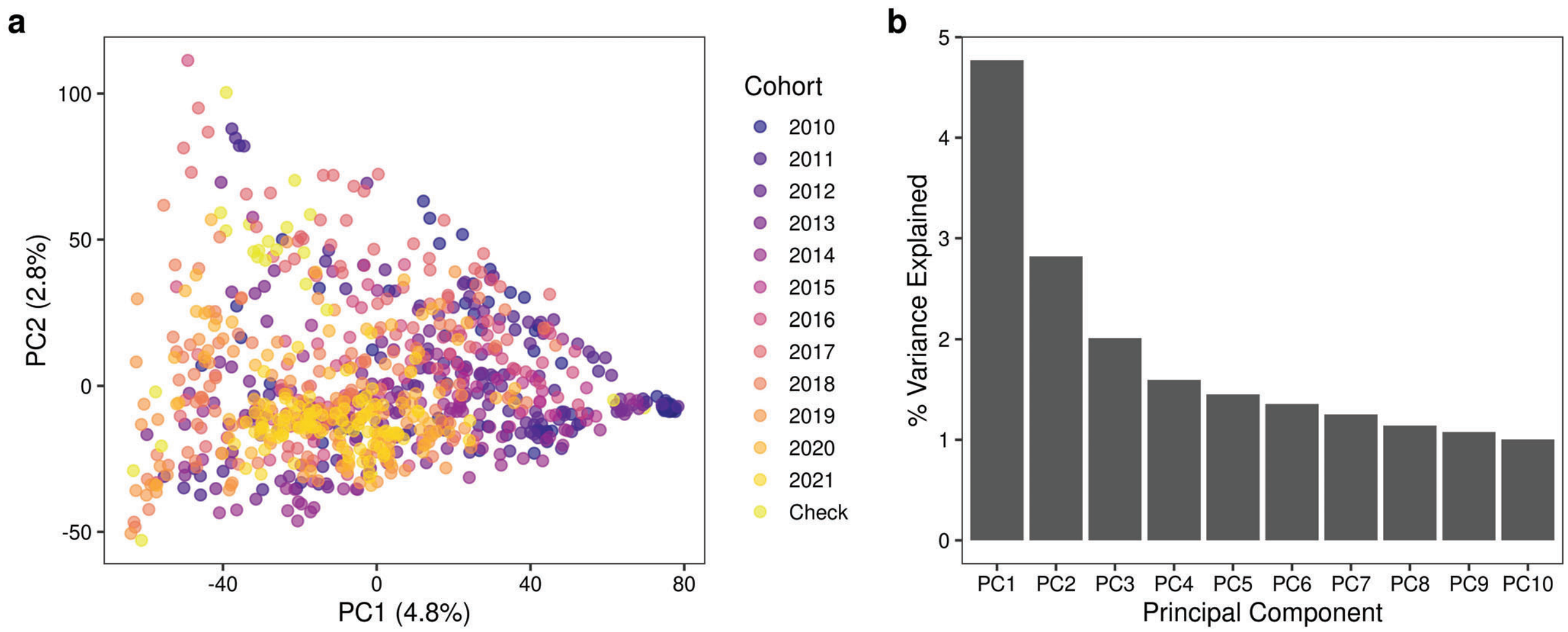

Supplementary Figure 2 a) Plot of principal components 1 and 2 derived from a principal component analysis (PCA) of the genetic marker matrix. The percentage of variance explained by each component is provided in parentheses. Points represent genotyped elite lines and are colored by the first year of phenotyping for each line. b) Scree plot of the same PCA data showing the percentage of the total variance explained of the first ten principal components.
